# Tick-Borne Pathogens in Ticks Collected from Wild Ungulates in North-Eastern Poland

**DOI:** 10.3390/pathogens10050587

**Published:** 2021-05-11

**Authors:** Mirosław M. Michalski, Katarzyna Kubiak, Magdalena Szczotko, Małgorzata Dmitryjuk

**Affiliations:** 1 Department of Parasitology and Invasive Diseases, Faculty of Veterinary Medicine, University of Warmia and Mazury in Olsztyn, 10–719 Olsztyn, Poland; michmm@uwm.edu.pl; 2 Department of Medical Biology, Collegium Medicum, School of Public Health, University of Warmia and Mazury in Olsztyn, 10–561 Olsztyn, Poland; katarzyna.kubiak@uwm.edu.pl; 3 Department of Biochemistry, Faculty of Biology and Biotechnology, University of Warmia and Mazury in Olsztyn, 10–719 Olsztyn, Poland; magdalena.szczotko@uwm.edu.pl

**Keywords:** *Borrelia burgdorferi* sensu lato, *Borrelia miyamotoi*, *Anaplasma phagocytophilum*, *Rickettsia* spp., tick-borne pathogens, *Ixodes ricinus*, *Dermacentor reticulatus*, wild mammals

## Abstract

This study was carried out in north-eastern Poland during two hunting seasons between 2018 and 2020. Ticks (*Ixodes ricinus* and *Dermacentor reticulatus*) were removed from wild cervids and boars and examined for the presence of *Borrelia* spirochetes and Rickettsiales members: *Rickettsia* spp. and *Anaplasma phagocytophilum*. The present study contributes to the knowledge of even-toed ungulates, which are an important reservoir of the above-mentioned pathogens and a potential source of infections for humans through ticks as vectors. Almost 40% of the collected ticks (191 out of 484) were infected with the following pathogens: 3.3% with *Borrelia* spp., 19.2% with *A. phagocytophilum* and 26.9% with *Rickettsia* spp. Only the ticks collected from cervids carried *Borrelia*. Typing of the species DNA confirmed the presence of *B. afzelii*, *B. garinii*, *B. lusitaniae* and *B. miyamotoi*. An analysis of *Rickettsia* spp. sequences using the GenBank data revealed the presence of *R. helvetica*, *R. raoultii* and *R. monacensis*. Monoinfections (79.1%) dominated over co-infections (20.9%). Among co-infections, the most frequent was *A. phagocytophilum*/*Rickettsia* spp. (70%), however co-infections, including *B. afzelii*/*A. phagocytophilum*, *B. afzelii*/*Rickettsia* spp., *B. miyamotoi*/*A. phagocytophilum* and *B. afzelii*/*B. garinii*/*B. lusitaniae*, were also noted. Significant differences were observed in the affinity of some pathogens to their vectors. Thus, *Borrelia* spp. and *A. phagocytophilum* were more frequently detected in *I. ricinus* (5.3% and 23.1%) than in *D. reticulatus* (1.2% and 15.3%). Infection frequency with *Rickettsia* spp. was similar (approximately 25–29%) in both tick species. The prevalence of *A. phagocytophilum* and *Rickettsia* spp. in ticks removed from cervids was 19.8% and 27.1%, and in ticks from wild boars it was 13.3% and 24.4%, respectively.

## 1. Introduction

Understanding the interrelationship between wildlife, livestock and public health presents a great challenge. Man-made landscapes, a mix of natural habitats and farmland, modify the abundance, composition and cohesion of populations and allow for contact between wildlife, livestock and humans. This, in turn, favors pathogen circulation among many hosts [1,2]. Wild animals, even at very low population densities, are important drivers of the abundance of ticks, as they promote their reproduction and passively spread potentially infected ticks. Viruses, bacteria and protozoa in ticks may be pathogenic to both humans and domesticated animals [3,4]. Therefore, the screening of wild animals, such as cervids and wild boars, is essential for revealing the relationship between sylvatic and domestic pathogen cycles. It provides basic information for a risk assessment of animal and human health in a given region [5]. Human interference in the natural ecosystems changes the populations of wild animals, as well as of ticks and the pathogens they transmit. The risk of tick-borne pathogen (TBP) transmission is increasing all over the world [6,7].

Serious diseases, including Lyme borreliosis (LB), anaplasmosis and tick-borne rickettsioses are caused by various bacterial species from the genera *Borrelia*, *Anaplasma* and *Rickettsia*. Recently, new pathogens are appearing, such as the spirochete *B. miyamotoi* that causes a relapsing fever “*Borrelia miyamotoi* disease” [8,9,10]. Different *Borrelia* species (or genotypes) cause varying LB clinical symptoms. Every year, the LB incidence in north-eastern Poland is almost twice the level of the rest of Poland [11]. In general, in Europe, small and medium-sized rodents, several species of birds, reptiles and insectivores are considered as competent reservoir hosts, i.e., those that may be infected with *Borrelia* and transmit the pathogen to uninfected ticks. In contrast, large wild and domestic ruminants (e.g., deer and sheep) are considered incompetent reservoirs since ticks feeding on them do not transmit *Borrelia*. However, cervids are an important source of blood in all stages of tick development and ticks may transmit spirochetes to each other when they feed very closely on an incompetent host [12].

Another very common tick-transmitted pathogen, *A. phagocytophilum,* causes human granulocytic anaplasmosis (HGA). Wild animals play a significant role in the prevalence and transmission of this pathogen. Scientific evidence has been accumulating on wildlife acting as a reservoir and an amplification “hub” of human or domestic animal diseases [13,14]. In Europe, cervids are suspected of being the main reservoir of *A. phagocytophilum*, with infection rates as high as up to 90% [2,15,16,17,18,19,20]. Variants detected in roe deer have been shown to be non-pathogenic to humans, dogs, horses and domestic ruminants, but variants detected in red deer and wild boars may cause HGA [17,18,19,20]. Cervids seem to play a more important role in the circulation of the *A. phagocytophilum* genetic variants pathogenic to humans and/or domestic and companion animals than wild boars [21].

The pathogenic potential of the genus *Rickettsia* is less clear. Among several *Rickettsia* classifications, the most common system uses four groups: spotted fever group (SFG), typhus group, the *Rickettsia bellii* group and the *Rickettsia canadensis* group [22]. In central Europe, the following five *Rickettsia* species have been detected in ticks: *R. slovaca*, *R. raoultii*, *R. helvetica*, *R. monacensis* and *Candidatus* Rickettsia mendelii [9]. *R. slovaca* and *R. raoultii* cause tick-borne lymphadenopathy (TIBOLA) and DEBONEL lymphadenopathy (*Dermacentor*-borne necrosis erythema). In contrast, although the pathogenic potential of *R. helvetica* and *R. monacensis* is controversial, they may cause a mild illness similar to spotted-fever-like disease [22]. Many *Rickettsiae* are transmitted vertically among ticks, which suggests that ticks are not only vectors but also reservoirs of rickettsiae in nature. However, these pathogens were also detected in numerous vertebrate hosts such as birds, reptiles and mammals [22,23].

This study aimed to compare the prevalence of particular tick-transmitted *Borrelia* spirochetes and Rickettsiales members: *Rickettsia* spp. and *A. phagocytophilum* in *I. ricinus* and *D. reticulatus* ticks removed from wild ungulates (cervids and boars) in north-eastern Poland during two hunting seasons between 2018 and 2020.

## 2. Results

### 2.1. Tick Collection

Among the 484 ticks collected from cervids (red deer, *Cervus elaphus* and roe deer, *Capreolus capreolus*) and wild boars (*Sus scrofa*) from three subregions (western, central and eastern) of northeastern Poland between 2018–2020, *I. ricinus* (*n* = 242, 50%) and *D. reticulatus* (*n* = 242, 50%) were identified (Table 1). A total of 242 of *I. ricinus* were removed from cervids (208 females, 33 males and 1 nymph) (*n* = 220, 91%) and wild boars (*n* = 22, 9%). Out of 242 *D. reticulatus* (50 females and 192 males) 90% (*n* = 219) were collected from cervids and 10% (*n* = 23) from wild boars. In the east, the most forested subregion of north-eastern Poland, 72.7% of the tested tick specimens originated from hunted wild ungulates. From western and central subregions 15.3% and 12% of all ticks were collected, respectively.

### 2.2. Prevalence and Diversity of Pathogens

The DNA of one, two or three tick-borne microorganisms was detected in 39.5% (191/484) of tick genomic DNA isolates. Overall, the prevalence of examined pathogens was 3.3% (*n* = 16) for *Borrelia* spp., 19.2% (*n* = 93) for *A. phagocytophilum* and 26.9% (*n* = 130) for *Rickettsia* spp. (Table 1). Among positive PCR products, 54% (129/239) originated from *I. ricinus* and 46% (110/239) from *D. reticulatus.* The highest percentage of ticks infected with bacteria *Borrelia* spp. and *A. phagocytophilum* was recorded in the central subregion of northeastern Poland (8/58, 13.8% and 17/58, 29.3%, respectively). Infections of *Rickettsia* spp. (29.0%, 102/352) were the most frequent in ticks feeding on wild ungulates from the eastern subregion (Table 1). 

#### 2.2.1. *Borrelia* spp.

A fragment of the *flaB* gene of *Borrelia* spp. was detected in 16 (3.3%) tick isolates, collected only from cervids (16/439, 3.6%) (Table 1). Most of them (13/16, 81.3%) were identified in *I. ricinus*. The *I. ricinus*-infection rate (13/242, 5.4%) was significantly higher (χ^2^ = 6.4, *p* < 0.05) than that in *D. reticulatus* (3/242, 1.2%). A comparison indicates that *I. ricinus* females harbored *Borrelia* spp. more often (6.0%, 13/215) (Table 1) than *D. reticulatus* males, as well (1.6%, 3/192) (*I. ricinus* females vs *D. reticulatus* males: χ^2^ = 5.4, *p* < 0.05). *I. ricinus* male and *D. reticulatus* female were not infected with *Borrelia* spirochetes.Species typing performed on the basis of RFLP patterns revealed the presence of DNA of *B. afzelii*, *B. garinii*, *B. lusitaniae* and *B. miyamotoi*. DNA of *B. afzelii*, the most dominant species, was detected in 11 samples from *I. ricinus* and in three samples from *D. reticulatus*. In one DNA sample from *I. ricinus*, *B. afzelii* was in co-infection with *B. garinii* and *B. lusitaniae*. *B. miyamotoi* was found in only one *I. ricinus* female tick (1/484, 0.2%). The highest percentage of ticks infected with the *Borrelia* genus derived from *I. ricinus* was recorded in the central subregion of north-eastern Poland (8/58, 13.8%) (Table 1) (central vs west: χ^2^ = 6.7; central vs east: χ^2^ = 16.5; *p* < 0.05).

**Figure 1:**
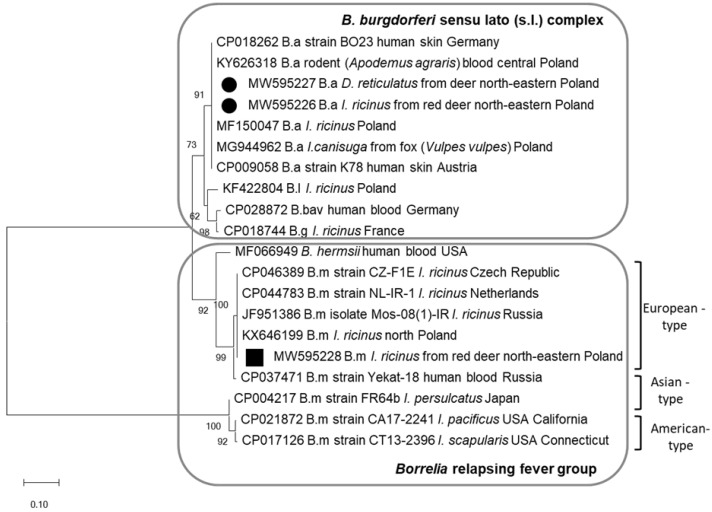
Molecular relationships between *Borrelia* species identified in the study and accession numbers from GenBank, based on the sequences of the *flaB* gene. Phylogram constructed using the neighbor-joining method and the Maximum Composite Likelihood as a distance method. Numbers at the tree nodes indicate the percent of bootstrap value from 1000 replicates. The tree is drawn to scale, with branch lengths measured in the number of base substitutions per site. The analyses were conducted in MEGA X. The sequences obtained in this study were labelled with black symbols. Abbreviations: B.a—*B. afzelii*, B.g—*B. garinii*, B.l—*B. lusitaniae*, B.bav—*B. bavariensis,* B.m—*B. miyamotoi.*

Three randomly selected PCR products identified by RFLP as *B. afzelii* and *B. miyamotoi* were sequenced. Sequences of *B. afzelii* (GenBank: MW595226–595227) were identical and showed 100% nucleotide identity with those of the BO23 and K78 strains, which are pathogenic to humans (GenBank: CP018262, CP009058) (Figure 1). These sequences were also 100% identical to *B. afzelii* sequences derived from *Ixodes* ticks questing (GenBank: MF150047) and feeding on fox (*Vulpes vulpes*) (GenBank: MG944962) and from the blood of rodent *Apodemus agrarius* (GenBank: KY626318) in Poland. The PCR product from *I. ricinus* removed from red deer skin identified as *B. miyamotoi* (GenBank: MW59528) was grouped with the sequence of isolate Mos-80 of *B. miyamotoi* from Russian *I. ricinus* designated as European-type [24,25]. On the phylogram, the obtained *B. miyamotoi* sequence clustered with other sequences derived from questing *I. ricinus* from the Czech Republic (GenBank: CP046389), from the Netherlands (GenBank: CP044783), and from Poland (GenBank: KX646199) (Figure 1).

#### 2.2.2. *Anaplasma phagocytophilum*

*A. phagocythophilum* DNA was identified in 19.2% (93/484) of tick DNA samples. The infection rate was significantly higher (χ^2^ = 4.8, *p* < 0.05) in *I. ricinus* (56/242, 23.1%) than in *D. reticulatus* (37/242, 15.3%) (Table 1). *Anaplasma* DNA was more frequently detected (χ^2^ = 10.4, *p* < 0.05) in *I. ricinus* females (23.7%, 51/215) in comparison with females of *D. reticulatus* (4%, 2/50). The percentage of infected *I. ricinus* and *D. reticulatus* males was similar (χ^2^ = 0.02, *p* = 0.9015) and reached 19.2% (5/26) and 18.2% (35/192), respectively. The highest percentage of ticks infected with *A. phagocytphilum* was detected in the central subregion of north-eastern Poland (17/58, 29.3%) (Table 1) (central vs. west: χ^2^ = 4.1, *p* < 0.05; central vs. east: χ^2^ = 3.7, *p* = 0.06).

Five positive PCR products amplified from DNA samples of different species of ticks collected from different hosts were sequenced and registered in GenBank under accession numbers MW591521–591525. All of them were identical and showed 100% similarity to the sequence of Norway variant2 of *A. phagocytophilum* (GenBank: CP015376) derived from sheep, isolate P13016 from the blood of a patient in Austria (GenBank: KT454992), and sequences from the blood of game animals in Portugal (GenBank: LC126876) and Romania (GenBank: KT351866).

#### 2.2.3. *Rickettsia* spp.

*Rickettsia* spp. was detected in 26.9% (130/484) of analyzed tick DNA samples. There were no significant differences in infection rate (χ^2^ = 1.05, *p* = 0.31) between *D. reticulatus* (70/242, 28.9%) and *I. ricinus* (60/242, 24.8%) (Table 1). The highest percentage of infected ticks was recorded among males of both species, i.e., 34.6% (9/26) for *I. ricinus* and 30.2% (58/192) for *D. reticulatus* (χ^2^ = 0.2, *p* = 0.65). Females of both tick species displayed similar levels (χ^2^ = 0.01, *p* = 0.91) of *Rickettsia* spp. infection, i.e., 23.3% (50/215) and 24% (12/50), respectively (Table 1). The highest *Rickettsia* spp. prevalence was noted in the eastern subregion of northeastern Poland (29.0%, 102/352) (Table 1). However, no statistically significant differences were noted (east vs central: χ^2^ = 2.2, *p* = 0.13; east vs west: χ^2^ = 0.9, *p* = 0.34).

**Figure 2:**
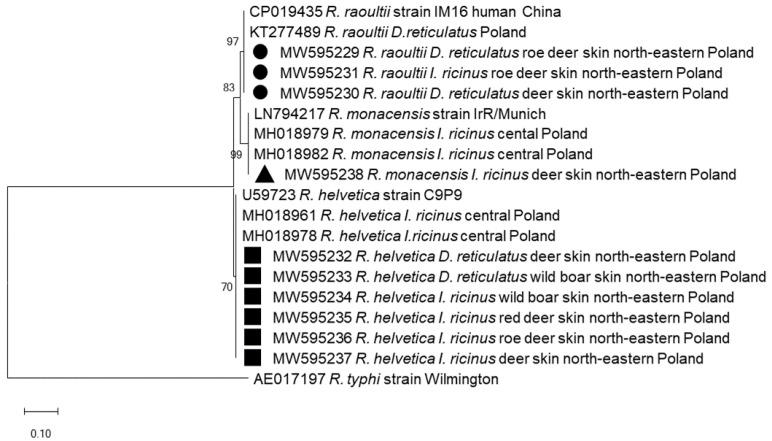
Molecular relationships between *Rickettsia* species identified in the study and accessions from GenBank, based on the sequences of the *gltA* gene of *Rickettsia.* Phylogram constructed using the neighbor-joining method and the Maximum Composite Likelihood as a distance method. Numbers at the tree nodes indicate the percent of bootstrap value from 1000 replicates. The tree is drawn to scale, with branch lengths measured in the number of base substitutions per site. The analyses were conducted in MEGA X. The sequences obtained in this study are labelled with black symbols.

To identify the *Rickettsia* species, ten amplicons of the *gltA* gene fragment (769 bp) were sequenced. Sequenced amplicons were derived from diverse tick species collected from different hosts. Comparison with the data registered in the GenBank revealed the presence of *R. helvetica* (*n* = 6), *R. raoultii* (*n* = 3) and *R. monacensis* (*n* = 1). Four *R. helvetica* sequences (GenBank: MW595234–595237) derived from *I. ricinus* were collected from red deer, roe deer and wild boar and two (GenBank: MW595232–595233) were derived from *D. reticulatus* feeding on deer and wild boar. All obtained *R. helvetica* sequences were identical and clustered with the *R. helvetica* strain C9P9 (GenBank: U59723) and sequences derived from questing *I. ricinus* from Poland (GenBank: MH018961–78) (Figure 2). Three sequences (MW595229–595231) derived from *I. riciuns* and *D. reticulatus* feeding on deer were identical with the *R. raoultii* strain IM16 isolated in a human sample from China (GenBank: CP019435) and from questing *D. reticulatus* in Poland (GenBank: KT277489) (Figure 2). The remaining single *Rickettsia* sequence (MW595238) obtained from *I. ricinus* collected from deer displayed 100% identity with *gltA* sequences of the *R. monacensis* strain IrR/Munich from Germany (GenBank: LN794217) and from questing *I*. *ricinus* ticks from Poland (MH018979–82).

#### 2.2.4. *Borrelia* spp., *Anaplasma phagocytophilum* and *Rickettsia* spp. Co-Infections

Monoinfections were identified in 79.1% (151/191) of tick DNA samples that were PCR-positive for microorganisms (monoinfections vs co-infections: χ^2^ = 129, *p* < 0.05). Among monoinfections, the most numerous were *Rickettsia* spp. (96/151, 63.6%) and *A. phagocytophilum* (52/151, 34.4%), while the least numerous were *B. afzelii* monoinfections (3/151, 2.0%) (*Rickettsia* spp. vs *A. phagocytophilum*: χ^2^ = 25.7, *p* < 0.05; *Rickettsia* spp. vs *B. afzelii*: χ^2^ = 122.9, *p* < 0.05). 

Co-infections of at least two pathogens were recognized in 20.9% (40/191) of positive samples (Table 2). The most frequently recorded co-infections were *A. phagocytophilum*/*Rickettsia* spp. (28/40, 70%, 95% CI: 53.4−83.4%). It was the only co-infection recorded in all three subregions. 

However, in the western subregion, results indicated a moderated positivity to *A. phagocytophilum* and *Rickettsia* spp., in correlation with each other (*r* = 0.4, *p* < 0.01). In the eastern direction positive results were more randomly distributed (central subregion: *r* = 0.08, *p* = 0.58; eastern subregion: *r* = 0.01, *p* = 0.91).

This tendency was not observed for other double co-infections (Table 2). Four cases of co-infection were recorded for *B. afzelii*/*A. phagocytophilum* (4/40, 10%, 95% CI: 2.8–23.6%), and *B. afzelii*/*Rickettsia* spp. (4/40, 10%, 95% CI: 2.8–23.6%). DNA of all three microorganism was detected in two ticks (2/40, 5%, 95% CI: 0.6–16.9). One co-infection was recorded with *B. miyamotoi*/*A. phagocytophilum* (1/40, 2.5%, 95% CI: 0.06–13.1%). One co-infection with three genotypes within the *B. burgdorferii* s.l. complex, i.e., *B. afzelii*/*B.garinii*/*B. lusitaniae*, was also recorded (1/40, 2.5%, 95% CI: 0.06–13.1%) (Table 2). 

More co-infections were detected in *I. ricinus* (28/40, 70.0%, 95% CI: 53.5–83.4) than in *D. reticulatus* ticks (12/40, 30.0%, 95% CI: 16.6–46.5) (Table 2). The result was significant at *p* < 0.5 (χ^2^ = 12.8, *p* < 0.05). Moreover, there were no statistically significant differences for the occurrence of mono-infections in both species of ticks (χ^2^ = 0.33, *p* = 0.56).

### 2.3. Comparison of Infection in Ticks from Different Hosts

All 16 cases of *Borrelia* spp. were detected in *I. ricinus* and *D. reticulatus* collected from cervids (16/439, 3.6%, 95% CI: 2.1–5.8%). Ticks removed from wild boars were not infected with *Borrelia* spirochetes. The prevalence of *A. phagocytophilum* and *Rickettsia* spp. in ticks feeding on cervids was 19.8% (87/439, 95% CI: 16.2–23.9%) and 27.1% (119/439, 95% CI: 23.0–31.5), while in ticks feeding on wild boars it was 13.3% (6/45, 95% CI: 5.0–26.8%) and 24.4% (11/45, 95% CI: 12.9–39.5), respectively. For both pathogens, infection rate was similar (for *A. phagocytophilum*: χ^2^ = 1.1, *p* = 0.29; for *Rickettsia* spp. χ^2^ = 0.1, *p* = 0.7). The infection rate in ticks removed from cervids and wild boars was also similar (χ^2^ = 0.3, *p* = 0.57).

## 3. Discussion

Depending on the daily rhythm of foraging and resting, and on the density of vegetation, free-living ungulates can cover long distances each day. This behavior makes them a readily accessible host for ticks which, in turn, are a reservoir of serious pathogens. Thus, cervids and boars contribute to the circulation of TBPs in the wilderness [26]. In the presented study, DNA of *Borrelia* spp., *A. phagocytophilum* and *Rickettsia* spp. was identified in ticks collected from ungulates hunted during two seasons in northeastern Poland.

The ticks collected in the current study represented two species: *I. ricinus* and *D. reticulatus*, which is similar to studies on ticks isolated from dairy cows in the eastern Poland [27]. From an epidemiological point of view, *I. ricinus* tick has greater importance than *D. reticulatus*. Although the *D. reticulatus* can transmit *Babesia canis*, bacteria of the genera *Rickettsia* and *Anaplasma*, or tick-borne encephalitis virus to the host [28], the involvement of this tick as a vector of *Borrelia* spirochetes is still unproven. *Borrelia* DNA in this tick species has already been reported. It should be noted that the region of northeastern Poland is considered typical of *D. reticulatus* and should be treated as a contiguous area of eastern populations of this tick [29]. 

Only limited data are available on *Borrelia* spirochete in ticks isolated from wild animals [6,30,31,32,33]. In the current study, the *Borrelia* infection rate was low, i.e., 3.3%, which is in line with the 3.4% reported from Spain [32]. In north-eastern Poland, for comparison, 31.6% of ticks isolated from dogs in urban areas, and 27.4% of questing ticks were infected with *Borrelia* [11,34]. The low level of *Borrelia* in the ticks found on wild mammals may be due to natural host immunity, involving an alternative complement activation in ungulate blood. These complements effectively eliminate *Borrelia* spp. from ungulates, and even from their ticks. Ticks feeding on red deer and wild boars have been shown to lose *Borrelia* infections [30]. Most ticks in this analysis were infected with *B. afzelii*, which is similar to the results reported by Seo et al. [6]. In Spain, *B. garinii*, *B. valaisiana*, *B. lusitaniae*, and *B. afzelii* were the most prevalent species in ticks removed from roe deer [32]. In the present study, *B. garinii* and *B. lusitaniae* were also detected in fully-engorged *I. ricinus* females isolated from red deer in the central part of north-eastern Poland (Bartoszyce hunting region), but those species occurred in a triple co-infection with *B. afzelii*. This study also confirmed the presence of *B. miyamotoi*, classified as a relapsing fever *Borrelia*. This indicates the constant presence of this pathogen in *I. ricinus* in Poland [10,11,35,36,37]. *B. miyamotoi* was also identified in two males and one nymph caught on a roe deer in Spain [32]. These results show that although deer may have limited utility as an indicator of the presence of *Borrelia* spirochetes, they provide for a good estimate of the species diversity of these TBPs in Europe. The contribution of *I. ricinus* to the spread of *Borrelia* is undeniable. Moreover, the present study detected *Borrelia* DNA in three *D. reticulatus* ticks. This seems surprising from the point of view of the existence of spirochete-eliminating mechanisms in ungulates and the presence of defensins in the salivary glands of *D. reticulatus*, which are attributed the role of specific antibiotics [38].

Ticks feeding on wild even-toed ungulates which were examined in this study were infected with *A. phagocytophilum* in a similar percentage to ticks isolated from wild cervids in west-central Poland. Both studies are also linked by the fact that female *I. ricinus* were infected more frequently with *A. phagocythophilum* than male ticks of this species [39]. In other studies in Poland, the rate of tick infection with *A. phagocytopilum* depends on the region and host species. In north-western Poland, ticks feeding on wild game were not infected with *A. phagocytophilum* [40], while in other studies from this region, ticks collected from roe deer and red deer were infected almost half as often as in the current research [15]. This study also suggests that at least in north-eastern Poland, wild mammals are probably an important reservoir of human anaplasmosis pathogen, i.e., *A. phagocythophilum*. To fully confirm this, however, it would be necessary to study the genetic variants of the pathogen. The obtained results are in agreement with previously published data in Europe [15,16,19,39,41,42,43,44] based on which rather convincing molecular evidence was presented last year on ticks isolated from urban dogs [34].

The present paper reports on the presence of *R. helvetica*, *R. monacensis,* and *R. raoultii* in ticks isolated from wild ungulates. The first two species have already been found in Poland in questing ticks and in ticks removed from Shetland ponies, cats, dogs, bats, and rodents [23,31,45,46,47]. *R. helvetica* caused only relatively mild symptoms in humans: a headache, sometimes a rash and an inoculation eschar. Infection with *R. monacensis* has a similar course, but in addition to inoculation eschar and a rash on hands and soles, it may cause flu-like symptoms and fever [22]. *R. raoultii* is similar to *R. slovaca* since it causes a more severe disease than the previous two species, i.e., TIBOLA or DEBONEL [22]. In Poland to date, *R. raoultii* has been identified mainly in *D. reticulatus* ticks [45,48,49,50,51,52,53]. This study also identified *R. raoultii* in *I. ricinus*, similar to Chmielewski et al. [45] who detected this pathogen in *I. ricinus* ticks collected from dogs and cats in central Poland.

A quantitative analysis of the data indicates that wild even-toed ungulates, along with other mammals, are important reservoirs of pathogens belonging to the SFG rickettsiae. In this study, 26.8% of ticks collected from wild ungulates were infected with SFG. For comparison, in 2015, 44% of adult questing ticks examined in the same area were infected [49]. In other studies in Poland, the degree of tick infection by *Rickettsia* spp. oscillates widely between 1.3% and 53% in questing ticks [23,45,48,50,51,52,54,55] and in ticks removed from different species of mammals, the tick infection rate ranges from 5.2% to 37.5% [31,46,47,56]. The present results are similar to those obtained in Germany, where approximately 23% of *Rickettsia* DNA-positive samples were found in ticks isolated from wild animals [57]. In addition, in this study, mono-infections account for nearly 80% of positive cases. However, among co-infections, 70% were the combination of *A. phagocytophilum*/*Rickettsia* spp. In the present study, a distinct west-east gradient emerges in the occurrence of this co-infection. While in the western region *A. phagocytophilum* and *Rickettsia* spp. are clearly positively correlated, in the regions towards the east they are increasingly randomly distributed. This might be connected with the increasing representation of *D. reticulatus* towards the east, which is endemic for this tick species, or with a greater dispersion of sampling sites in the east of the region. The frequent coexistence of both pathogens along with their relatively high prevalence in ticks studied in the current study, confirms that cervids and wild boars are important reservoirs of Rickettsiales bacteria. 

In summary, it should be emphasized that although cervids have limited utility as an indicator of the presence of *Borrelia* spirochetes, they may provide a good estimate of the species diversity of these TBPs in Europe. In northeastern Poland, wild even-toed ungulates, along with other mammals, are reservoirs of pathogens belonging to SFG rickettsiae and another member of Rickettsiales: *A. phagocytophilum*. 

## 4. Materials and Methods

### 4.1. Study Area, Tick Collection and Species Identification

Ticks were collected from red deer (*C. elaphus*), roe deer (*C. capreolus*) and wild boar (*S. scrofa*) during two consecutive legal hunting seasons (September–April) between 2018–2020. The hunted animals came from nine hunting districts located in the west (Ostróda), central (Bartoszyce, Reszel, Gietrzwałd, Olsztynek) and east (Kowale Oleckie, Giżycko, Pisz, Bielsk Podlaski) subregions in northeastern Poland (Warmia and Mazury province). The hunters isolated one to seven ticks per animal and then preserved the collected ticks in 70% ethanol. In the laboratory, the species, developmental stage and sex of the ticks were identified using taxonomic keys [58]. 

### 4.2. DNA Extraction

Before DNA extraction, ticks preserved in 70% ethanol were dried. Full-engorged ticks were bisected along their longitudinal axis to provide the optimal weight of each sample. Anterior parts of fully-engorged or whole non- and slightly-engorged ticks were separately crushed using a sterile mortar. The material was then transferred to 2 mL tubes filled with lysis buffer (A&A Biotechnology, Gdynia, Poland) and incubated for 2 h at 50 °C. After lysis, total DNA was extracted according to the manufacturer’s protocol (Micro AX Tissue Gravity, A&A Biotechnology, Gdynia, Poland) and stored at −70 °C until further analyses.

### 4.3. Pathogens DNA Detection 

#### 4.3.1. *Borrelia* Species

The presence of *Borrelia* DNA in ticks was confirmed by the nested polymerase chain reaction method using two sets of primers specific to the flagellin gene (*flaB*): outer—132f/905r (774 bp) and inner—220f/823r (604 bp) [35] (Table 3). The 25 μL of PCR mixture contained 12.5 μL of DreamTaq Green PCR Master MIX (Thermo Scientific, Waltham, MA, USA), 10.5 μL nuclease-free water, and 0.5 μL of each primer (10 μM), and 1 μL of template DNA or 1 μL of the outer PCR product for nested PCR. The thermal conditions of PCRs were as follows: 2 min at 94 °C, by 40 cycles of 30 s at 94 °C, 30 s at 50 °C (54 °C for inner primers), 1 min at 72 °C. Finally, an extension step of 1 min was performed at 72 °C. 

To identify the *Borrelia* species, the restriction fragment length polymorphism (RFLP) method was used [59]. The positive inner PCR products (604 bp) were digested with restriction endonuclease *Hpy*F3I (Fast Digest Tsp 509I, Thermo Scientific, Waltham, MA, USA) according to the manufacturer’s instructions. Restriction fragments were separated on 3% agarose gel and stained with Midori Green dye (Nippon Genetics Europe GmbH, Düren, Germany). The obtained RFLP patterns enabled the identification of nine *Borrelia* species: *B. garinii*, *B. afzelii*, *B. burgdorferi* sensu stricto (s.s.), *B. lusitaniae*, *B. valaisiana*, *B. bissetti*, *B. spielmanii*, *B. bavariensis* and *B. miyamotoi* including the RF group of *Borrelia* [35,59].

#### 4.3.2. *Anaplasma phagocytophilum*

*A. phagocytophilum* DNA was detected using the EHR521 and EHR747 primers [60] (Table 3) targeting a fragment of the 16S rRNA gene (247 bp) according to the protocol described by Michalski et al. [34].

#### 4.3.3. *Rickettsia* Species

To detect and identify species of *Rickettsia* spp., primers CS409 and Rp1258 were used for the amplification of 769 bp fragment of the *gltA* gene [61] (Table 3). The 25 μL of PCR mixture contained 12.5 μL of DreamTaq Green PCR Master MIX (Thermo Scientific, Waltham, MA, USA), 5.5 μL nuclease-free water, 1 μL of each primer (10 μM) and 5 μL of template DNA using the following PCR thermal conditions: 5 min at 95 °C, by 40 cycles of 30 s at 94 °C, 30 s at 55 °C, 55 s at 72 °C and a final extension of 7 min at 72 °C. 

#### 4.3.4. PCRs

All PCRs were carried out using a Mastercycler Nexus (Eppendorf, Hamburg, Germany). PCR products were visualized by electrophoresis on 1.5% agarose gel stained with Midori Green DNA dye (Nippon Genetics Europe GmbH, Düren, Germany). Each PCR analysis included negative (nuclease-free water instead of DNA) and positive control samples. The positive controls were a commercial sample of *B. burgdorferi* s.l. DNA (DNA Gdańsk, Poland) and samples of DNA positive for *A. phagocytophilum* and *Rickettsia* spp. obtained from ticks which were purified and confirmed by sequencing. 

### 4.4. DNA Sequencing and Data Analysis

A randomly selected representative number of PCR products positive for *Borrelia* spirochetes (*n* = 3), *A. phagocytophilum* (*n* = 5) and *Rickettsia* spp. (*n* = 10) were purified using the Clean Up purification kit (A&A Biotechnology, Gdynia, Poland) according to the manufacturer’s protocol and bidirectionally sequenced at Macrogen Europe (Amsterdam, The Netherlands). The obtained nucleotide sequences were edited in BioEdit software [62] and compared with data registered in the GenBank database (http://www.ncbi.nih.gov/Genbank/index.html, accessed on: 11 February 2021) using the BLAST-NCBI program (http://www.ncbi.nlm.nih.gov/BLAST/, accessed on: 9 April 2021). Consensus sequences of the fragment of *Borrelia flaB* gene were deposited in the GenBank database and registered under the accession numbers: MW595226–595228, for the *A. phagocytophilum* 16S RNA gene: MW591521–591525 and the *Rickettsia gltA* fragment gene: MW595229–595238.

Representative *Borrelia* and *Rickettsia* sequences obtained in this study and the most similar sequences chosen from GenBank were used in phylogenetic analysis. The phylogram was constructed using a method based on the neighbor-joining method and the Maximum Composite Likelihood as a distance method. The topology of the phylogram was evaluated using the bootstrap method with 1000 replicates. Phylogenetic analysis was conducted using MEGA X software (Penn. State University, Philadelphia, PA, USA). 

### 4.5. Statistical Analysis

Each tick was analyzed individually. The prevalence was expressed using percentages. A statistical analysis of the results was performed using a two-sided Fisher’s exact test (Prism 6 program, GraphPad Software, San Diego, CA, USA). The prevalence of pathogens was calculated with 95% confidence intervals (95% CI) using the "exact" interval by Clopper and Pearson. A Chi-square test (χ^2^) was used to check whether there was a relationship between variables, i.e., a prevalence of pathogens in both tested tick species and between subregions. Pearson’s correlation coefficient (*r*) was determined to establish the association of double co-infections with the subregions. Values of *p* < 0.05 were considered statistically significant. 

## 5. Conclusions

Pathogens of the complex of *B. burgdorferi* s.l. (*B. afzelii*, *B. garinii*, *B. lusitaniae*), *B. miyamotoi* relapsing fever spirochete, *A. phagocytophilum*, and *Rickettsia* spp. have been detected in ticks removed from wild even-toed ungulates in north-eastern Poland. The low level of spirochete infections among ticks removed from wild mammals can be explained by the loss of *Borrelia* infections during feeding. The relatively high infestation level of ticks feeding on wild mammals, especially cervids, and infected with *A. phagocytophilum* and spotted fever rickettsiae group, may indicate that these animals are important reservoirs of both pathogens, contributing to their circulation in nature and are a potential source of infections for humans through ticks as vectors. This study illustrates that wild ungulates with heavy tick exposure in north-eastern Poland can be infected more frequently with single than multiple tick-borne pathogens of potential clinical importance.

## Figures and Tables

**Table 1:** Infection rates of ticks removed from wild ungulates in north-eastern Poland.

Subregion	Hosts	Tick Species and Sex ^1^		*Borrelia* spp. No. %, (95% CI)	*Anaplasma* spp. No. %, (95% CI)	*Rickettsia* spp. No. %, (95% CI)
West	Cervids	*I. ricinus*	F	1/63 1.6 (0.04–8.5)	9/63 14.3 (6.7–25.4)	12/63 19.0 (10.2–30.9)
			M	0/11 0.0 (0.0–28.5)	2/11 18.2 (2.3–51.7)	5/11 45.5 (16.7–76.6)
		Subtotal		1/74 1.4 (0.03–7.3)	11/74 14.9 (7.6–25.0)	17/74 23.0 (14.0–34.2)
Central	Cervids	*I. ricinus*	F	8/56 14.3 (6.4–26.2)	17/56 30.4 (18.8–44.1)	11/56 19.7 (10.2–32.4)
		*D. reticulatus*	F	0/1 0.0 (0.0–97.5)	0/1 0.0 (0.0–97.5)	0/1 0.0 (0.0–97.5)
	Wild Boar	*I. ricinus*	F	0/1 0.0 (0.0–97.5)	0/1 0.0 (0.0–97.5)	0/1 0.0 (0.0–97.5)
		Subtotal		8/58 13.8 (6.1–25.4)	17/58 29.3 (18.1–42.7)	11/58 19.0 (9.9–31.4)
East	Cervids	*I. ricinus*	F	4/80 5.0 (1.4–12.3)	21/80 26.3 (17.0–37.3)	24/80 30.0 (20.2–41.3)
			M	0/10 0.0 (0.0–30.8)	1/10 10.0 (0.25–44.5)	2/10 20.0 (2.5–55.6)
		*D. reticulatus*	F	0/45 0.0 (0.0–7.8)	2/45 4.4 (0.5–15.1)	9/45 20.0 (9.6–34.6)
			M	3/173 1.7 (0.3–5.0)	35/173 20.2 (14.5–27.0)	56/173 32.4 (25.4–40.0)
	Wild Boar	*I. ricinus*	F	0/15 0.0 (0.0–21.8)	4/15 26.7 (7.8–55.1)	3/15 20.0 (4.3–48.1)
			M	0/5 0.0 (0.0–52.2)	2/5 40.0 (5.2–85.3)	2/5 40.0 (5.2–85.3.0)
			N	0/1 0.0 (0.0–97.5)	0/1 10.0 (0.0–97.5)	1/1 100.0 (2.5–100)
		*D. reticulatus*	F	0/4 0.0 (0.0–60.2)	0/4 0.0 (0.0–60.2)	3/4 75.0 (19.4–99.3)
			M	0/19 0.0 (0.0–17.6)	0/19 0.0 (0.0–17.6)	2/19 10.5 (1.3–33.1)
		Subtotal		7/352 2.0 (0.8–4.0)	65/352 18.5 (14.5–22.9)	102/352 29.0 (24.3–34.0)
Subtotal Sex		*I. ricinus*	F	13/215 6.0 (3.2–10.1)	51/215 23.7 (18.2–30.0)	50/215 23.3 (17.8–29.5)
			M	0/26 0.0 (0.0–13.2)	5/26 19.2 (6.5–39.3)	9/26 34.6 (17.2–55.60
			N	0/1 0.0 (0.0–97.5)	0/1 10.0 (0.0–97.5)	1/1 100.0 (2.5–100)
		*D. reticulatus*	F	0/50 0.0 (0.0–7.1)	2/50 4.0 (0.5–13.7)	12/50 24.0 (13.0–38.1)
			M	3/192 1.6 (0.3–4.5)	35/192 18.2 (13.0–24.4)	58/192 30.2 (23.8–37.2)
Total Species and Sex				16/484 3.3 (1.9–5.3)	93/484 19.2 (15.8–23.0)	130/484 26.9 (22.9–31.0)

^1^ F—female, M—male, N—nymph.

**Table 2:** Co-infection rates of ticks removed from wild mammals in the hunting subregions of northeastern Poland, with the pathogens of the genera *Borrelia*, *Anaplasma* and *Rickettsia*.

Subregion	Host ^1^	Tick Species ^2^	Double Co-Infections No. Pearson’s Correlation for Subregions				Triple Co-Infections No.	
			*Ap*/*R*	*Ba*/*Ap*	*Ba*/*R*	*Bm*/*Ap*	*Ba*/*Ap*/*R*	*Ba*/*Bg*/*Bl*
West	C	*Ir*	7/74 *r* = 0.4, *p* < 0.01 ^3^	0/74	0/74	0/74	0/74	0/74
Central	C	*Ir*	4/56	0/56	2/56	1/56	2/56	1/56
		*Dr*	0/1	0/1	0/1	0/1	0/1	0/1
	WB	*Ir*	0/1	0/1	0/1	0/1	0/1	0/1
	Subtotal Central		4/58 *r* = 0.08, *p* = 0.58	0/58	2/58 *r* = 0.06, *p* = 0.65	1/58 *r* = 0.21, *p* = 0.12	2/58	1/58
East	C	*Ir*	7/90	2/90	1/90	0/90	0/90	0/90
		*Dr*	9/218	2/218	1/218	0/218	0/218	0/218
	WB	*Ir*	1/21	0/21	0/21	0/21	0/21	0/21
		*Dr*	0/23	0/23	0/23	0/23	0/23	0/23
	Subtotal East		17/352 *r* = 0.01, *p* = 0.91	4/352 *r* = 0.14, *p* < 0.01	2/352 *r* = –0.0004, *p* = 1	0/352	0/352	0/352

^1^ C—cervids, WB—wild boar; ^2^ Ir—*Ixodes ricinus*, Dr—*Dermacentor reticulatus*; Ap—*Anaplasma phagocytophilum*, R—*Rickettsia* spp., Ba—*Borrelia afzelii*, Bm—*Borrelia miyamotoi*, Bg—*Borrelia garinii*; Bl—*Borrelia lusitaniae*; ^3^ r—Pearson’s correlation coefficient.

**Table 3:** Primer sets used for PCR amplification.

Primer Name	Primer Sequence 5’—3’	Product Size [bp]	Species Gene	References
132f	TGGTATGGGAGTTCTGG	774	*Borrelia* spp. *flaB* ^1^	[35]
905r	TCTGTCATTGTAGCATCTTT			
220f	CAGACAACAGAGGGAAAT	604		
823r	TCAAGTCTATTTTGGAAAGCACC			
EHR521	TGTAGGCGGTTCGGTAAGTTAAAG	247	*A. phagocytophilum* 16S rRNA	[60]
EHR747	GCACTCATCGTTTACAGCGTG			
CS409	CCTATGGCTATTATGCTTGC	769	*Rickettsia* spp. *gltA* ^2^	[61]
Rp1258	ATTGCAAAAAGTACAGTGAACA			

^1^*flaB*- flagellin gene, ^2^*gltA-* citrate synthase gene.

## Data Availability

The data presented in this study are contained within the article.
